# Supplementary material for: Carbon content and other soil properties of near-surface peats before and after peatland restoration
Source: PeerJ. 2024 Apr 18;12:e17113. doi: 10.7717/peerj.17113 (PMC11032657; doi:10.7717/peerj.17113)
Supplement: Supplemental Information 1 [file peerj-12-17113-s001.docx]

**Readme** for rawdata of the article „Carbon content and other soil properties of near-surface peats before and after peatland restoration“

################

General description:

In contrast to the text, commas are used as decimal separators in all tables of the raw data.

Additional remark: The CSV files use ISO-8859-1 (Latin 1) encoding for text.

################

Table: Statistics_R_Stechlin_final.csv

| parameter | unit | datatype | description |
| --- | --- | --- | --- |
| site_number | - | number (integer) | specific number for each site (site code) |
| site | - | string | site name |
| sampling | - | number (integer) | sampling intervall number (intervall code) |
| sampling_1 | - | string | sampling intervall |
| site_number_sampling | - | number (integer) | code for site and sampling intervall first number: site code second number: intervall code |
| profile | - | string | code of specific sampling point |
| new_peat | cm | number (integer) | thickness of new peat above old surface layer |
| TOC | % | number (numeric) | total organic carbon content |
| CN | - | number (numeric) | ratio of TOC/TN |
| TN | % | number (numeric) | total nitrogen content |
| pH | - | number (numeric) | pH-value |
| DBD | g/cm³ | number (numeric) | dry bulk density |

################

Table: peat_old_new.csv

| parameter | unit | datatype | description |
| --- | --- | --- | --- |
| site_number | - | number (integer) | specific number for each site (site code) |
| site | - | string | site name |
| old_new_peat | - | string | new – new peat above old topsoil old – old topsoil layer |
| old_new_number | - | number (integer) | 1 – new peat above old topsoil 2 – old topsoil layer |
| site_number_old_new | - | number (integer) | code for site and new/old peat first number: site_number second number: old_new_number |
| profile | - | string | code of specific sampling point |
| thickness_new | cm | number (integer) | thickness of new peat above old surface layer |
| TOC | % | number (numeric) | total organic carbon content |
| CN | - | number (numeric) | Ratio of TOC/TN |
| TN | % | number (numeric) | total nitrogen content |
| pH | - | number (numeric) | pH-value |
| DBD | g/cm³ | number (numeric) | dry bulk density |

################

Table: Statistics_waterlevel.csv

| parameter | unit | datatype | description |
| --- | --- | --- | --- |
| month | - | date | date of waterlevel measurement |
| site_number | - | number (integer) | specific number for each site (site code) |
| site | - | string | site name |
| waterlevel | cm | number (numeric) | water level related to surface („-„ below surface, „ „ above surface) |

################

Table: statistics-differences-sampling.csv

| parameter | unit | datatype | description |
| --- | --- | --- | --- |
| site | - | number (integer) | specific number for each site (site code) |
| Site_name | - | string | site name |
| TOC | % | number (numeric) | differences between first sampling and second sampling period of total organic carbon content |
| CN | - | number (numeric) | differences between first sampling and second sampling period of ratio of TOC/TN |
| TN | % | number (numeric) | differences between first sampling and second sampling period of total nitrogen content |
| pH | - | number (numeric) | differences between first sampling and second sampling period of pH-value |
| DBD | g/cm³ | number (numeric) | differences between first sampling and second sampling period of dry bulk density |
